# The improving care in chronic obstructive lung disease study: CAROL improving processes of care and quality of life of COPD patients in primary care: study protocol for a randomized controlled trial

**DOI:** 10.1186/1745-6215-15-96

**Published:** 2014-03-27

**Authors:** Claudia Steurer-Stey, Stefan Markun, Kaba Dalla Lana, Anja Frei, Ulrike Held, Michel Wensing, Thomas Rosemann

**Affiliations:** 1 Institute of General Practice and Health Services Research, University of Zurich, Pestalozzistrasse 24, 8091 Zurich, Switzerland; 2 Scientific Institute for Quality in Healthcare, Radboud University Nijmegen Medical Center, Nijmegen, Netherlands

## Abstract

**Background:**

The Swiss health ministry launched a national quality program ‘QualiCCare’ in 2011 to improve health care for patients with COPD.

The aim of this study is to determine whether participation in the COPD quality initiative (‘QualiCCare’) improves adherence to recommended clinical processes and shows impact on patients’ COPD care and on the impact of COPD on a person's life.

**Methods:**

CAROL is a cluster-randomized controlled trial with randomization on the general practioner (GP) level. Thirty GPs will be randomly assigned to equally sized intervention group or control group.

Each GP will approach consecutively and regardless of the reason for the current consultation, patients aged 45 years or older, with a smoking history of ≥ ten pack-years (PY). Patients with confirmed (by spirometric evaluation) COPD will be included in the study. GPs in the intervention group will receive ‘QualiCCare’ education, which addresses knowledge, decision-making and behavioural aspects as well as delivery of care according to COPD quality indicators and evidence-based key elements. In the control group, no educational intervention will be applied and COPD patients will be treated as usual. The study period is one year.

The primary outcome measure is an aggregated score of relevant clinical processes defining elements in the care of patients with COPD: smoking cessation counseling, influenza vaccination, motivation for physical activity, appropriate pharmacotherapy, patient education and collaborative care. Given a power of 90% and a significance level alpha of 5%, 15 GPs recruiting eight patients each will be necessary in both study arms. With an assumed dropout rate of 20%, 288 patients will need to be included.

**Discussion:**

It is important to develop and implement interventions that add value to COPD care considering quality and efficiency. Care pathways modifying the knowledge and behavior of physicians have the potential for improving care by transferring knowledge to clinical practice.

**Trial registration:**

ClinicalTrials.gov: NCT01921556

## Background

**Figure 1:**
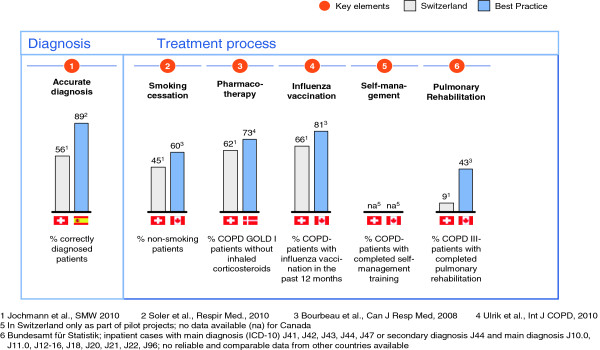
Differences in six chronic obstructive pulmonary disease (COPD) key elements between Swiss primary care and published international data.

Chronic obstructive pulmonary disease (COPD), an illness with a prevalence of about 10% in the adult Swiss population causes significant burden to patients and the health care system [1-3]. COPD often remains undiagnosed, and if diagnosed, attention is mainly directed towards pharmacologic treatment, in particular treatment of exacerbations [4]. The course of illness and quality of life of patients can be improved by pharmacological and non-pharmacological interventions [5,6]. Besides appropriate pharmacological treatment, smoking cessation, sustaining physical activity, influenza vaccination and empowering patients to recognize and self-manage exacerbations in an early phase define high quality care of patients with COPD. Recently, we reported on gaps in COPD management in Swiss primary care with overuse of inhaled corticosteroids in moderate COPD and too little emphasis on smoking cessation counseling and patient education [7] (Figure 1). Such deficiencies in health care delivery lead to increased morbidity and excessive use of health care resources [8] and disclose the need for quality improvement.

Studies in various countries have demonstrated that features of successful COPD care programs are the appropriate delivery of medication, planned regular consultations, activation and education of the patients, adequate exacerbation management and collaborative, integrated disease management [9-13]. The successful COPD programs used more or less a framework known as the chronic care model (CCM) [14] for developing and implementing activities to improve primary care for patients with chronic illnesses. The CCM combines delivery system redesign, clinical information systems, decision support, and self-management support within a practice, linked with health care organization and community resources beyond the practice. Successful implementation of CCM elements provided evidence for improved COPD care [4,15]. An important assumption of the CCM is that the effect of a number of elements and organizational factors combined is higher than their sum of effects.

In 2011, the Swiss health ministry launched the national quality initiative ‘QualiCCare’ to improve the quality of care for COPD patients. In relation to international data, it focuses on adherence with COPD guideline recommendations and on CCM based actions that emerged to improve the course of COPD by prevention and reduction of exacerbations, and thereby also health care costs [4-6,16,17]. These actions include a more proactive and coordinated COPD team care to provide self-management support, smoking cessation counseling, annual influenza vaccination, motivation for physical activity and pulmonary rehabilitation and appropriate pharmacologic treatment considering severity, symptoms and risk of exacerbations. The central aspect of the ‘QualiCCare’ program is a multifaceted implementation approach. It aims not only to increase knowledge but also internal motivation and decision-making by offering stimuli, resources and written instruments [18-21]. Further it categorizes and delineates specific professional and organizational interventions and comprises a short list of evidence-based practice items into a primary care ‘COPD care bundle’ that should guide and support better performance. So far, experience with care bundles for COPD only exists for discharge in patients hospitalized with COPD exacerbations [22].

In Switzerland, such a multifaceted COPD targeted quality program has not yet been broadly implemented and evaluated. The aim of the study is, therefore, to evaluate the effect of the ‘QualiCCare’ COPD intervention on COPD management in patients with diagnosed COPD in primary care practices in the Canton of Zurich.

### Hypotheses

The ‘QualiCCare’ intervention will improve adherence to suggested ‘good care’ standards in COPD, namely: smoking cessation counseling, influenza vaccination, appropriate pharmacotherapy with less overuse of inhaled steroids and correct inhalation technique, consideration of exacerbations, physical activity, pulmonary rehabilitation and patient education, and care coordination. Improvement in these processes is the first step towards improved clinical outcome measures as health status, and therefore, we also expect over time, a reduced impact of COPD on patients’ lives.

## Methods/Design

We will conduct a cluster-randomized controlled trial (randomization on GP level) with primary care physicians in the Canton of Zurich in the years 2013 and 2014.

### Recruitment and eligibility of primary care physicians

GPs are eligible for participation if they provide care in the routine primary setting (single handed practices and group practices) to unselected patients.

About 300 GPs in the area of Zurich and Winterthur are invited to an information meeting by a formal letter of the Institute of General Practice and Health Services Research of the University of Zurich and the Department of Health of the Canton of Zurich. In this information meeting an overview of the study will be given. The main focus of the first information meeting for all GPs is to present the project and to motivate GPs to take part in the study. Additionally, the project will be presented in several quality circle meetings in doctors’ networks (regions Zurich, Winterthur).

GPs who agree to participate will be randomized, stratified by practice organization (single handed versus group practices). All participating GPs/practice assistants will receive instruction by a pneumologist and a spirometry technician in performing a spirometry. Practices in the intervention group get detailed information on evidence-based COPD management and ‘QualiCCare’ training sessions and instruments, designed to increase knowledge, internal motivation and decision-making and to support incorporation of evidence into their daily work (details described in the intervention section).

The GPs of the control group apply ‘care as usual’ without receiving the ‘QualiCCare’ training and implementation tools.

### Patient recruitment

All GPs are asked to approach consecutively and regardless of the reason for the current consultation, patients aged 45 years or older with a smoking history of ≥ ten pack-years (PY) and to include patients with COPD diagnosed by spirometric evaluation.

To assure an appropriate spirometric evaluation, all GPs will participate in a spirometric training by a pneumologist and a spirometry technician before starting the patient recruitment process and before randomization of the GPs. The aim of this procedure is to diagnose COPD or confirm the diagnosis of COPD by a current and correct spirometry in both arms of the study.

### Patient inclusion criteria

1) Males and females ≥ 45 years of age and

2) Smoker or ex-smoker (with at least ten PY) and

3) Obstruction in spirometry FEV_1_/FVC < 0.7

### Patient exclusion criteria

1. No obstruction in spirometry (FEV1/FVC ≥ 0.7) or

2. Patients with history of asthma or hay fever or

3. Other concomitant pulmonary disease or

4. Patients with malignancies of any other system and/or other severe disease with an estimated life expectancy of less than six months or

5. Insufficient German language skills or

6. Patients who contact the practice for emergencies only or as a substitute practice

**Figure 2:**
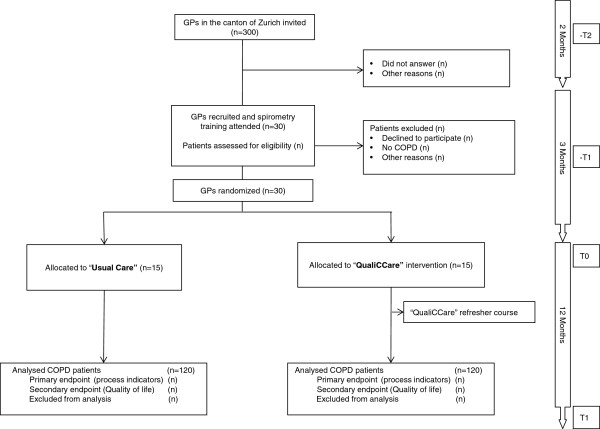
Study flow chart.

The flow chart of GP and patient recruitment of the study is shown in Figure 2.

### Clinical interventions/guideline adherence

With the ‘QualiCCare’ intervention, we reach out to implement the recommended key clinical processes in primary care for patients with diagnosed COPD namely: smoking cessation counseling, yearly influence vaccination, counseling to increase motivation for physical activity and pulmonary rehabilitation, self-management education with a written action plan to do the right thing at the right time in case of an exacerbation; appropriate pharmacologic treatment for stable and exacerbated COPD, and proactive, collaborative COPD care.

### Implementation program

#### Instruction of the GPs and practice assistants in the intervention group

Physicians randomized into the intervention group get a ‘QualiCCare’ training workshop designed to educate professionals on the guidelines, but also and particularly, governing professional behavior by feedback, reminders and pathways that help to change their attitudes and care behavior [23-25]. Based on behavioral and learning theory [26], ‘QualiCCare’ intervention not only tries to increase knowledge but also internal motivation and decision- making by stimuli and resources and by written instruments that guide evidence-based decision support.

The education sessions will actively involve GPs and practice assistants with tasks and responsibilities and also, by demonstrating them (by interactive teaching, case vignettes and role plays), ways on how evidence-based tasks could be incorporated into their daily work.

Three instruments (A-C) have been developed based on a review of international and national guidelines and in collaboration with the Swiss Respiratory Society, Swiss Societies of Internal and General Medicine and College of Primary Care Medicine, with a specific focus on primary care circumstances and needs. These instruments will be introduced to the intervention group as working tools for daily practice to support decision-making and adherence with recommended COPD care.

A. The Swiss COPD quintessence for primary care is a clinical guideline for GPs which is consistent with international guidelines for COPD [27] and the newly published Swiss COPD guideline [6], but more comprehensive and summarizing the key elements of COPD management for Swiss primary care [28].

B. The COPD pocket guide contains evidence-based information, assessment tools (MRC, CAT, STS), opinion sheets (early diagnosis, smoking cessation advice), treatment algorithm and decision aids for the key COPD elements: Confirm diagnosis, Optimize symptoms, Prevent deterioration, and Develop network and self-management support. The pocket guide also contains addresses and links to useful information as well as for specialist support, pulmonary rehabilitation and community resources offering, for example, smoking cessation and patient education.

C. The Primary care COPD Care bundle is something new for the primary care setting. So far, experience for COPD only exists for discharge care bundles for COPD patients hospitalized with acute exacerbations. The implementation of a discharge care bundle improved adherence with optimum health care practice [22]. Our primary care COPD care bundle comprises a short list of evidence-based practice items to be implemented into primary care for patients with COPD. It can function as a reminder and as a checklist of all necessary activities that can be ticked as performance control. It is expected to function as a stimulus for increased guideline adherence and internal motivation for behavior change.

Bundle items selected for primary care are:

1. Identify COPD in a smoker or ex-smoker (with at least ten PY) with symptoms (cough, sputum or dyspnea).

2. If the patient is still a smoker, offer smoking cessation assistance.

3. Motivate for physical activity and consider/refer for pulmonary rehabilitation.

4. Motivate for influenza vaccination.

5. Offer appropriate pharmacologic treatment for stable and exacerbated COPD.

6. Demonstrate and check correct use of inhalers.

7. Give written information about COPD including Lunge Zürich self-management booklet and an action plan.

8. Offer proactive follow-up and integrated services (for example follow-up, feedback and scheduling and ‘navigation’ functions by the trained practice assistant, arranging appointments, and connecting patients to internal and external providers and resources).

A specialized and experienced team of a pneumologist and a respiratory physiotherapist and master trainer will hold the ‘QualiCCare’ training workshop (half a day session). The three dimensions knowledge, skills and behavior in COPD management are addressed with consideration of the COPD care bundle items.

A ‘QualiCCare’ refresher course will be held six months after the first training workshop.

### Outcomes

#### Primary outcome

Difference in ‘quality of care process’ (total increase in performed measures/fulfilled indicators) after one year between COPD patients in the intervention and control group as reported by the patient (described below).

#### Secondary outcomes

a. Percentage of COPD patients with FEV1 < 60% and/or ≥ two exacerbations who received

• advice to participate in pulmonary rehabilitation

• referral to pulmonary rehabilitation

• a written action plan for exacerbation management

• proactive follow up and integrated services (arrangement for follow up and referrals)

b. Quality of life (CAT)

c. Aggregate measure for ‘quality of care process’ reported by the GPs

Details on the purpose defined, aggregate outcome measure (primary outcome and secondary outcome b):

Every process indicator will get a rating of 1, when performed (or done) and 0 when not performed. The range of the primary outcome will be from 0 to a maximum of 9, the range of the secondary outcome - applicable only for a subgroup of patients - will be 0 to a maximum of 13:

Indicators for the primary outcome:

1. Smoking cessation advice

2. Smoking cessation intervention

3. Influenza vaccination

4. Assessment of physical activity

5. Advice for physical activity

6. Assessment of exacerbation frequency

7. Appropriate pharmacological treatment: (no inhaled corticosteroids in patients with FEV1 > 50% and < two exacerbations/year)

8. Instruction in correct inhalation technique

9. Information and patient education: additional indicators for the secondary outcome: COPD patients with FEV1 < 50% or ≥ two exacerbations

10. A written action plan for exacerbation management

11. Advice to participate in pulmonary rehabilitation

12. Referral to pulmonary rehabilitation

13. Proactive follow-up and integrated services

#### Quality of life

We use the COPD Assessment Test (CAT). The CAT provides clinicians and patients with a reliable measure of overall COPD-related health status for the assessment and long-term follow-up of individual patients. The CAT is a validated short and simple patient-completed questionnaire that has been developed for use in routine clinical practice to measure the health status and grading the impact of COPD on patients’ life. The CAT is available in different languages (http://www.catestonline.org). It compromises eight simple questions, eight items on a scale of 0 to 5 with a total scoring range of 0 to 40; CAT score < 10 low impact, 10 to 20 medium impact and > 30 very high impact of COPD on a patient’s life [29].

### Measures at baseline and follow up

#### Baseline

Patient demographics: gender, age, race/ethnicity, employment status, and family background.

Clinical characteristics: comorbidities (predefined selection of frequent comorbidities cardiovascular, diabetes type II), BMI, smoking status, PYs smoking, respiratory symptoms cough, sputum, dyspnea and severity of dyspnea (Medical research council MRC dyspnea scale), spirometry (FEV1/FVC < 70%) and grade of severity (FEV1 > 80% GOLD I mild COPD, FEV1 50 to 80% GOLD II moderate COPD, FEV1 30 to 50% GOLD III severe COPD, FEV1 < 30% GOLD IV very severe COPD) according to GOLD guidelines. Burden of COPD in daily life assessed with the COPD assessment test (CAT). Current drug therapy, exacerbations and health care utilization in previous year due to pulmonary problems, including hospital admissions and emergency department attendances or unscheduled visits are also assessed.

### Follow-up

We use a data collection tool with clinical questionnaires for patients and GPs (each with an allocated corresponding code) to measure performance.

### Data collection procedures

#### Patient questionnaire

The patients will receive detailed written information on the aim of the study. After giving their written informed consent they will receive a questionnaire (T0) assessing gender, age, employment status, and family background, the CAT, information on respiratory symptoms (cough, sputum, dyspnea), and severity of dyspnea (Medical research council MRC dyspnea scale), smoking status, motivation to quit, exacerbations and health care utilization (including unscheduled visits, emergency department attendances and hospital admissions due to pulmonary problems). Patients are asked at T0 whether, in the previous year before study inclusion, they had received: advice to quit smoking, assessment and advice for physical activity or advice/referral to pulmonary rehabilitation, recommendation/application of influenza vaccination, patient education with defining COPD and information about the main causes, signs and symptoms, information on medication and using inhalation devices with face-to-face instruction and demonstration of inhalation technique, information on how to identify and manage an aggravation of symptoms (exacerbation) and an action plan for exacerbation management including medication adjustment for exacerbation treatment. In addition patients are asked whether they have received COPD support by community resources (lung associations, support groups) and other specialized health professionals. Patients are asked the same questions one year after study inclusion T1. They receive the questionnaires and stamped envelopes with the postal address of the university and are asked to return the questionnaire in the envelope to the university at T0, and T1 after 12 months.

Patients will also be informed that neither the GP nor the practice team has any possibility of obtaining knowledge of their answers.

#### GPs

The GP will maintain a record of the participants with an allocated code for each patient. This code is also marked on the questionnaires. The university only receives the patients’ codes and has no access to their names.

A questionnaire is filled out by the GPs with information on the professional characteristics (gender, age, specialty, work time) and of the organizational context, such as single or group practice, regional structures and available resources.

At T0, the GP gives information for each participating patient on demographics, clinical characteristics: comorbidities, BMI, smoking status, PYs smoking, information on respiratory symptoms cough, sputum, dyspnea and severity of dyspnea (Medical Research Council MRC dyspnea scale) spirometry (FEV1/FVC < 0.7) and grade of severity according to GOLD guidelines FEV1 > 80% GOLD I mild COPD, FEV1 50 to 80% GOLD II moderate COPD, FEV1 30 to 50% GOLD III severe COPD, FEV1 < 30% GOLD IV very severe COPD), exacerbation frequency and current (pulmonary) drug therapy.

After 12 months (T1), GPs are asked about smoking cessation counseling activities, yearly influenza vaccination and assessment of physical activity; if ‘yes’ , how was physical activity assessed? Possibilities include anamnesis and objective tests, for example, sit to stand test (STS), six-minute walking test, and ergometer. Also asked about are motivation for physical activity and referral to pulmonary rehabilitation, assessment of exacerbation frequency and health care utilization in previous year, including hospital admissions, emergency department attendances and unscheduled visits. Whether patient education activities are given, verbally and/or via patient booklet, with definition of COPD and information about the main causes, signs and symptoms of COPD is also inquired into, as is whether information on medication and using inhalation devices, face-to-face instruction and demonstration of inhalation technique is supplied. Additionally, whether information on how to identify and manage an aggravation of symptoms (exacerbation) and whether an action plan for exacerbation management, including a written medication adjustment for exacerbation treatment is supplied, is asked about. GPs are also asked whether they educate patients in the practice or refer them to an external COPD program connecting COPD patients with specialized health professionals and community resources (lung associations, support groups).

This procedure will be carried out to compare patients’ reported procedures with GPs’ self-reported performed procedures and is assessed as secondary outcome measure.

The GP questionnaires are also marked with the patient’s code and will be returned to the university in a stamped envelope. GP measurements will take place at baseline (pre-data collection) and after 12 months (post-data collection). Pre-data collection is estimated to last three months.

An independent research assistant of the university will enter the data directly into the SPSS program (version 18.0 or higher, SPSS, Chicago, IL, USA)**.**

For an intermediate outcome, we will assess mediating processes with a self-efficacy scale for professional knowledge, skills and behaviour before and right after the ‘QualiCCare’ workshop and after 12 months.

The process evaluation will also include outreach visits and interviews, which have shown to be effective strategies to support implementations in primary care. Therefore, one outreach visit will be performed eight to twelve weeks after the training workshop for GPs and practice assistants in the intervention group. The aim of this outreach visit is to assess if the structures in the practices are appropriate to perform care according to the study protocol. Furthermore, the visit aims to reveal possible problems which might have occurred, and to discuss and implement appropriate solutions. Whether the instruments are used as intended will also be checked. The outreach visit will be performed by a study nurse of the study center.

### Statistical analysis

#### Calculation of sample size

Based on available data from Switzerland [7,30], and a recently performed cross-sectional survey of our study group, we assume a mean of four regarding the number of performed indicators (primary outcomes 1 to 9 as mentioned above) and a SD of 2.3. We assume an increase in the intervention group in the mean of performed indicators of 1.5. Given a power of 90% and a significance level alpha of 5%, as well as an intracluster correlation coefficient (ICC) of 0.04, we will need eight patients per GP assuming participation of 30 GPs, resulting in 240 patients. Assuming a dropout rate of at least 20% (dropouts are possible on a GP level but also on a patient level, therefore this rate is estimated to be about 20%), we will need 288 patients.

#### Data analysis

A *t*-test will be used for independent group comparisons in the primary outcome between control and intervention group. Each quality indicator itself can also be regarded as a binary outcome (performing a specific procedure (yes/no)). To analyze these binary outcomes separately logistic regression will be used to model the relationships between the outcome and treatment group, age and gender. Other potentially important covariates will be identified through exploratory analyses. The longitudinal aspect of the data can be incorporated into the model in various ways; we will utilize the generalized estimating equations (GEE) approach [31].

For continuous outcomes such as quality of life (QoL) instruments, repeated measures analysis of variance is appropriate [32].

Fixed effect parameters will include treatment group, age, gender, and other potentially important covariates. The primary data analysis will follow the intent-to-treat (ITT) approach where appropriate. This means that all available data from all individuals will be analyzed according to treatment group assignment, regardless of whether or not each individual actually received the assigned treatment.

### Timeframe of the study

The recruitment of the 30 GPs is planned over two months between July and August 2013 (−T2).

Patient eligibility screening and patient inclusion is projected within a period of three months (−T1). The intervention courses will start subsequently.

Assessments will be made as described above, T0 baseline assessments start from the inclusion; at baseline, data regarding COPD management in the year before inclusion will also be assessed retrospectively from the patient charts and transferred anonymously to the study center.

Final measurements (T1) will be performed one year after the ‘QualiCCare’ intervention.

### Ethical principles

The study is conducted in accordance with medical professional codex and the Helsinki Declaration as of 1996 as well as Data Security Laws and according to good clinical practice guidelines.

Study participation of patients is voluntary and can be cancelled at any time without provision of reasons and without negative consequences for their future medical care.

### Patient informed consent

Previous to study participation, patients receive written and verbal information about the content and extent of the planned study from the GPs; for instance about potential benefits and potential risks to their health. In case of acceptance, they sign the informed consent form.

In case of study discontinuation, all material will be destroyed or the patient will be asked if he/she accepts that existing material can be analyzed in the study.

### Vote of the Ethics Committee

The study protocol is approved by the Ethics Committee of the Kanton Zurich (reference KEK-ZH_number 2013-0189).

### Data security/disclosure of original documents

The patient names and all other confidential information fall under medical confidentiality rules and are treated according to appropriate Federal Data Security Laws. The results of the patient questionnaires are not accessible to the GPs. Questionnaires are directly mailed to the study center by the patient.

All study related data and documents are stored on a protected central server of the University of Zurich. Only direct members of the internal study team can access the respective files.

Intermediate and final reports are stored in the office of the Department of General Practice and Health Services Research at the Zurich University Hospital (USZ).

## Discussion (expected results)

Improving care for people with chronic diseases is one of the big challenges in health care worldwide [17]. In this era plagued by ever-tightening health care resources, it is of utmost importance to identify and implement interventions that are of added value to quality and efficiency of care [14,33]. Efficient professional interventions and care pathways, which detail essential steps in the care of patients, have the potential for improving patient care by reaching quality standards and decreasing unwarranted practice variation. However, evaluation of their impact is important. According to Lemmens *et al*. [34] improvement of expertise, information and resources will affect behavioral intention, which leads to professional behavioral change and this should lead to improved health effects. We expect that the ‘QualiCCare’ intervention can be implemented into primary care practices and will constitute a path forward for better quality in COPD care. After adjusting for age, gender, number of chronic conditions, participating sites are expected to show significant improvement for several quality indicators, including smoking cessation advice, influenza vaccination, appropriate medical therapeutics, rehabilitation and self-management education efforts and better proactive, collaborative care compared to the ‘usual care’ group.

Professional support and improvement in these processes is the first step towards improved clinical outcome measures as quality of life and therefore, we also expect a significant difference for our secondary outcome QOL assessed by the patient.

In addition, we try to identify professional behavior change mechanisms and gain insight into some implementation processes that could help to open the ‘black box’ that frequently lies between input and outcome of interventions.

The vision that emerges from our study is of organizations that aim to implement best practice and to improve the value of care in COPD, one of the world’s major chronic illnesses. This study is, therefore, of great interest not only from the patient perspective but also from a health care system perspective.

### Trial status

Patient recruitment had not started at the time of first submission in August 2013.

## Abbreviations

BMI: body mass index; CAT: COPD assessment test; CCM: chronic care model; COPD: chronic obstructive pulmonary disease; FEV1: forced expiratory volume in first second; FVC: forced vital capacity; GEE: generalized estimating equations; GOLD: Global initiative on Obstructive Lung Disease; GP: general practitioner; ICC: intracluster correlation coefficient; ICS: inhaled corticosteroids; ITT: intent-to-treat; MRC: Medical Research Council dyspnea scale; PY: pack-years; QoL: quality of life; RCT: randomized-controlled trial; STS: sit to stand test.

## Competing interests

The authors declare that they have no competing interests.

## Authors’ contributions

CS: conception and design, financial support, data collection and analysis, manuscript writing and final approval of the manuscript. SM: data collection and analysis, critical revision and final approval of the manuscript. KD: data collection and analysis, critical revision and final approval of the manuscript. AF: data collection and analysis, critical revision and final approval of the manuscript. UH: statistics and analysis, critical revision and final approval of the manuscript. MW: conception and design, manuscript writing, final approval of manuscript. TR: conception and design, financial support, critical revision and final approval of the manuscript. All authors read and approved the final manuscript.
